# Epigenetic Variation in Monozygotic Twins: A Genome-Wide Analysis of DNA Methylation in Buccal Cells

**DOI:** 10.3390/genes5020347

**Published:** 2014-05-05

**Authors:** Jenny van Dongen, Erik A. Ehli, Roderick C. Slieker, Meike Bartels, Zachary M. Weber, Gareth E. Davies, P. Eline Slagboom, Bastiaan T. Heijmans, Dorret I. Boomsma

**Affiliations:** 1 Department of Biological Psychology, VU University Amsterdam, Van der Boechorststraat 1, 1081 BT Amsterdam, The Netherlands; E-Mails: m.bartels@vu.nl (M.B.); di.boomsma@vu.nl (D.I.B.); 2 Avera Institute for Human Genetics, 3720 W. 69th Street, Sioux Falls, SD 57108, USA; E-Mails: Erik.Ehli@avera.org (E.A.E.); Zachary.Weber@avera.org (Z.M.W.); Gareth.Davies@avera.org (G.E.D.); 3 Department of Psychiatry, University of South Dakota, 4400 W. 69th Street, Sioux Falls, SD 57108, USA; 4 Department of Molecular Epidemiology, Leiden University Medical Center, P.O. Box 9600, 2300 RC Leiden, The Netherlands; E-Mails: R.C.Slieker@lumc.nl (R.C.S.); P.Slagboom@lumc.nl (P.E.S.); B.T.Heijmans@lumc.nl (B.T.H.)

**Keywords:** DNA methylation, cytosine, epigenetics, Illumina 450k, twin study, buccal epithelium, genome-wide methylation, children

## Abstract

DNA methylation is one of the most extensively studied epigenetic marks in humans. Yet, it is largely unknown what causes variation in DNA methylation between individuals. The comparison of DNA methylation profiles of monozygotic (MZ) twins offers a unique experimental design to examine the extent to which such variation is related to individual-specific environmental influences and stochastic events or to familial factors (DNA sequence and shared environment). We measured genome-wide DNA methylation in buccal samples from ten MZ pairs (age 8–19) using the Illumina 450k array and examined twin correlations for methylation level at 420,921 CpGs after QC. After selecting CpGs showing the most variation in the methylation level between subjects, the mean genome-wide correlation (rho) was 0.54. The correlation was higher, on average, for CpGs within CpG islands (CGIs), compared to CGI shores, shelves and non-CGI regions, particularly at hypomethylated CpGs. This finding suggests that individual-specific environmental and stochastic influences account for more variation in DNA methylation in CpG-poor regions. Our findings also indicate that it is worthwhile to examine heritable and shared environmental influences on buccal DNA methylation in larger studies that also include dizygotic twins.

## 1. Introduction

To date, hundreds of genetic risk variants for complex traits and diseases have been identified, although for most of these variants, the biological mechanisms remain to be elucidated [1]. Interestingly, the majority of disease-associated genetic variation is located in regulatory regions of the genome [2], including transcription-factor-occupied regions and DNase I hypersensitive sites (which correspond to open chromatin) [3]. This suggests that mechanisms that control the activity of genes, including epigenetic mechanisms, may represent an important link between DNA sequence variation and common disease susceptibility [4]. Trying to unravel the molecular biology underlying complex traits and disease, much attention has been drawn recently to these epigenetic mechanisms; non-DNA sequence-based regulation of gene expression by DNA methylation, histone modification, microRNAs, *etc*. [5]. DNA methylation is one of the most extensively studied epigenetic mechanisms in human populations and tissues and is the focus of this paper.

In humans, DNA methylation occurs almost exclusively at cytosines that are part of CpG dinucleotides. The relationship between DNA methylation and expression varies depending on the genomic context: CpG methylation at promoter regions is generally thought to repress gene expression, while gene body methylation is generally associated with active gene expression and has been suggested to regulate splicing [6,7,8]. In most cell types, the majority of CpGs in the genome (on average, 70%–80%) is typically methylated [9]. Of the unmethylated CpG sites in the genome, most occur in areas of clustered CpGs, called CpG islands, which are often present in promoter regions. Yet, DNA methylation patterns may vary, and differential methylation has been demonstrated to occur across age [10], cell types, tissues [7,11] and disease states [12,13], and it has become clear that widespread variation in methylation patterns exist between individuals [14]. Accumulating evidence suggests that DNA methylation patterns can be affected by genetic variants (mQTLs) [15], environmental exposures [16] and stochastic factors [17,18], but it is largely unknown how much each of these factors account for overall variation between individuals in DNA methylation across the genome. Twin studies provide insight into the proportion of inter-individual variation in DNA methylation that is due to genetic variation, environmental effects and stochastic variation [19].

Because MZ twins derive from a single zygote and, therefore, have (nearly) identical DNA sequences (see, for example, Ye *et al.*, 2013 [20]), the comparison of DNA methylation patterns of MZ twins allows one to examine the extent to which differences in methylation between human individuals are related to environmental and stochastic events. Previous studies have highlighted that various tissues of MZ twins already show differences in DNA methylation at birth [21,22] and that differences between twins for average genome-wide DNA methylation, total histone acetylation levels and methylation at certain loci increase with age (referred to as “epigenetic drift”) [23]. Although a cross-sectional study of DNA methylation discordance in saliva from 34 MZ pairs (age range: 21–55 years) found no evidence for larger differences in DNA methylation in older MZ pairs [24], results from a cross-sectional analysis based on 230 MZ pairs (age range: 18–89 years) suggested a gradual increase of DNA methylation discordance in MZ twins from early adulthood to advanced age at various candidate loci, which was supported by longitudinal data from 19 elderly MZ pairs [25].

In the past few years, various studies have examined DNA methylation at a set of candidate genes or particular genomic regions in MZ and dizygotic (DZ) twins [26,27,28,29,30,31], usually reporting greater similarity of MZ twins compared to DZ twins, suggesting that heritable influences contribute to DNA methylation variation at specific regions. While CpG sites at some imprinted loci showed evidence for moderate to high heritability in blood samples from adolescent and middle-aged twins [29], other genomic regions, including the major histocompatibility complex (MHC) region, showed little evidence for genetic influences on DNA methylation variation [28]. Twin studies also highlighted variation between tissues in the importance of genetic influences on methylation of candidate loci at birth [30]. A longitudinal classical twin study of three candidate genes (*DRD4*, *SLC6A4/SERT* and *MAOA*) based on buccal cells indicated that changes in the methylation of these genes within individuals between age five and 10 are mostly attributable to non-shared environmental influences and stochastic variation [31]. Clearly, twin studies of candidate regions suggest that there is broad variation in the importance of heritable influences and environmental or stochastic variation to DNA methylation at different regions.

To date, only a few genome-scale analyses of DNA methylation have been performed using the classical twin design, including a study of ~12,000 CpG sites within islands [32], two studies that used a promoter-specific array targeting ~27,000 CpG sites (Illumina 27k) [21,33] and two studies that used the Infinium HumanMethylation450 array (Illumina 450k) [22,34], which assesses ~485,000 CpG sites across a variety of regions in the genome, including gene bodies and intergenic regions [35]. The studies that assessed heritability consistently reported that the average heritability of the methylation level at CpGs across the genome is low to moderate when all sites are considered, although the heritability of individual CpGs ranges between 0% and 100%. The following estimates of average heritability across genome-wide CpGs have been reported to date (based on all analyzed CpGs): 18% in blood from 32- to 80-year-old twins (21 MZ pairs and 31 DZ pairs) [33], 5% in placenta, 7% in human umbilical vascular endothelial cells (HUVEC) and 12% in cord-blood mononuclear cells (CBMC) from neonatal twins (22 MZ and 12 DZ pair [21]) and 19% in adipose tissue from adult female twins (97 MZ pairs and 162 DZ pairs) [34]. In two studies of neonatal twin tissues, methylation discordance in MZ and DZ twins increased with increasing distance from CpG islands (CGIs) for certain probes (Type I), *i.e.*, differences were larger in the shores and shelves that flank CGIs [21,22]. In the study of adipose tissue, it was noted that the average genome-wide heritability of DNA methylation was higher when restricting to the most variable CpG sites (for the top 10% CpGs of which the methylation level varied most between subjects, the average heritability was 37%) [34]. It was also found that gene body and intergenic regions showed higher average methylation levels, more variation between subjects and higher heritability compared to promoter regions in adipose tissue [34].

To summarize, there is great interest in unraveling the factors that contribute to variation in DNA methylation between persons, but most previous twin studies of DNA methylation have been limited to candidate genes or a subset of regulatory regions in the genome (mostly promoter regions and CGIs). Two earlier studies used the Illumina 450k to collect genome-wide data in MZ and DZ twins; one in adipose tissue in adults [34] and one in DNA isolated from buccal cells in infants (10 MZ pairs and five DZ pairs, longitudinal design) [22]. In line with earlier findings suggesting the divergence of DNA methylation profiles with age in MZ twins (mostly based on data from adult twins, cross-sectional comparisons and limited genomic coverage), Martino *et al.* [22] showed that widespread DNA methylation changes occur across the genome in buccal cells between birth and 18 months and that some MZ and DZ pairs already show divergence of DNA methylation profiles, whereas other pairs show stable difference levels or became more similar within the first 18 months after birth. In this paper, we analyzed genome-wide DNA methylation profiles (Illumina 450k) from buccal epithelium. We focused on 10 young and adolescent MZ twin pairs (age 8–19). The aim of our study was to examine how similar the DNA methylation profiles of buccal cells from genetically identical subjects are in childhood and adolescence and whether MZ twin similarity varies between different genomic regions.

Previous studies have highlighted differences in mean methylation level, differences in the effect of methylation level on gene expression and differences in the effect size and direction of the effect on methylation for disease associations across different regions in the genome [6]. These findings indicate that the establishment and maintenance of DNA methylation is differentially regulated in different regions and that a given change in methylation in different areas may have different downstream effects, suggesting that DNA methylation in some regions may be more tightly controlled than in others. We questioned whether these regional differences are also accompanied by differences in the importance of environmental and stochastic influences *versus* familial factors (genetic variation and shared environment) to inter-individual variation in methylation levels. Therefore, we describe the MZ twin correlations of individual CpGs as a function of various genomic classifications, including the position relative to CGIs (CGI regions, shores, shelves and non-CGI regions), genes (distal to promoter, proximal to promoter, gene body and intergenic) and ENCODE regulatory regions (DNaseI hypersensitive sites (DHS) and transcription factor binding sites (TFBS)). Hereby, our study gives valuable insight into the factors influencing inter-individual genome-wide DNA methylation variation in buccal cells in childhood and adolescence and into the degree to which these influences vary across functional regions in the genome.

## 2. Experimental

### 2.1. Subjects

Ten monozygotic twin pairs, who take part in longitudinal studies of the Netherlands Twin Register (NTR), were selected for the current study. There were five young twin pairs [36] whose buccal samples were collected when the twins were between ages 8 and 10 years and five adolescent pairs [37] who were aged 18–19 years at the time of sample collection. In the young group, there were three male pairs and two female pairs, and in the adolescent group, there were two male pairs and three female pairs. The twins were unselected with respect to phenotypic characteristics. Informed consent was obtained from the parents (children) or from the twins themselves (adolescents). The study was approved by the Central Ethics Committee on Research Involving Human Subjects of the VU University Medical Centre, Amsterdam, an Institutional Review Board certified by the U.S. Office of Human Research Protections (IRB number IRB-2991 under Federal-wide Assurance-3703; IRB/institute codes, NTR 03-180). Participants could indicate if they wished to be informed of the results of zygosity testing. Zygosity testing, based on a set of SNPs and VNTRs, as described in Van Beijsterveldt *et al.* 2013 [36], confirmed that all pairs were MZ. In addition to the twin samples, a single sample was used as a genomic DNA control. This DNA sample (CEPH) was derived from a stable cell line (female) from the HapMap project and was run in four replicates on the methylation BeadChip arrays.

### 2.2. Buccal DNA Collection

The procedures of buccal swab collection [38] and genomic DNA extraction [39] have been described previously. In short, 16 cotton mouth swabs were individually rubbed against the inside of the cheek by the participants and placed in four separate 15 mL conical tubes (four swabs in each tube) containing 0.5 mL STE buffer (100 mM sodium chloride, 10 mM Tris hydrochloride (pH 8.0) and 10 mM ethylenediaminetetraacetic acid) with proteinase K (0.1 mg/mL) and sodium dodecyl sulfate (SDS) (0.5%) per swab. Individuals were asked to refrain from eating or drinking 1 hour prior to sampling. High molecular weight genomic DNA was extracted from the swabs using a high salt (KAc) precipitation followed by a standard chloroform/isoamyl alcohol (24:1) extraction. The DNA samples were quantified using absorbance at 260 nm with a Nanodrop ND-1000 (Nanodrop Technologies, Wilmington, DE, USA).

### 2.3. Infinium HumanMethylation450 BeadChip Data Generation

The epigenome-wide methylation data was generated using the Infinium HumanMethylation450 BeadChip Kit (Illumina Inc., San Diego, CA, USA). The Infinium HumanMethylation450 BeadChip is able to interrogate over 450,000 methylation sites across the entire genome, including 99% of RefSeq genes. Content was selected to include gene regulatory regions, such as the promoter, 5' UTR, first exon, gene body and the 3' UTR. Additionally, bead probes were also designed to cover regions adjacent to the CpG islands, such as the shores and shelves [35].

The Infinium DNA methylation assay was performed at the Avera Institute for Human Genetics. The assay was completed exactly as denoted in the manufacturer’s protocol. The concentration of genomic DNA used in the Infinium DNA methylation assay was determined by comparing the binding of PicoGreen to known standards (λ DNA) and to the sample DNA. Briefly, 500 ng of genomic DNA was used for bisulfite conversion using the Zymo EZ DNA methylation kit (Zymo Research). Five microliters of bisulfite-converted DNA were whole genome amplified, which was followed by enzymatic end-point fragmentation. The resulting fragments were purified using an isopropanol precipitation, and the resuspended genomic DNA was denatured and hybridized to the BeadChip arrays for 18 hours. Extension, staining and washing were completed manually in flow cells followed by imaging using the iScan system (Illumina, Inc.). The raw data were extracted as *idat* files and were used in the downstream analysis.

### 2.4. Quality Control, Normalization and Data Processing

The raw intensity files (*idat*) were imported into the R environment [40], where further processing, quality control and normalization took place. The performance of bisulfite control probes confirmed successful bisulfite conversion for all samples. For each sample, we compared the overall (median) methylated signal intensity to the overall unmethylated signal intensity across all probes and compared the overall signal intensity from all CpG probes to the overall background signal (“noise”), as assessed using negative control probes. The overall signal from CpG probes was good and well-separated from the background signal for all samples. As a final quality check of the samples, cluster analysis was performed (cluster method = complete linkage) based on the Euclidean distance between samples, which was calculated from the pair-wise correlations between samples using the most variable probes (probes with an SD of the β-value across all 24 samples >0.10, with probes on the X and Y chromosomes and probes containing SNPs, as described in the next paragraph excluded; Nprobes = 38,359). The results of the cluster analysis were visualized in a dendrogram (see the “Results” section), which showed no outlier samples and illustrated tight clustering of the four replicate measures of control DNA.

Several probe-level QC steps were performed to filter out probes with low performance. For all samples, ambiguously-mapped probes were excluded, based on the definition of an overlap of at least 47 bases per probe from Chen *et al.* [41], and all probes containing an SNP, identified in the Dutch population [42], within the CpG site (at the C or G position) were excluded, irrespective of minor allele frequency. For each sample individually, probes with an intensity value of zero (not present on the array of a particular sample), probes with a detection *p-*value > 0.01 (calculated using the function *detectionP* from the *minfi* package) and probes with a bead count <3 were excluded. After these steps, probes with a success rate <0.95 across samples were removed from all samples, and the success rate across probes for each sample was computed (range of per sample success rate: 0.9990–0.9998).

After QC, background and red/green color adjustment were applied to the raw probe intensity values using quantile normalization. Normalized intensity values were converted into beta-values (β). The β-value, which represents the methylation level at a CpG for an individual and ranges from 0 to 1, is calculated as:

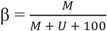

where M = methylated signal, U = unmethylated signal and 100 represents a correction term to control the β-value of probes with a very low overall signal intensity (*i.e.*, probes for which M + U ~ 0 after background subtraction).

Finally, in anticipation of our categorization of CpGs based on the mean β-value across samples, β-values were adjusted to account for (intra-sample) differences in the distributions of methylation values derived from Type I probes (two bead types per CpG site) *versus* Type II probes (one bead type per CpG site) using the beta-mixture quantile normalization method (BMIQ) [43].

### 2.5. Genomic Annotations

**Figure 1:**
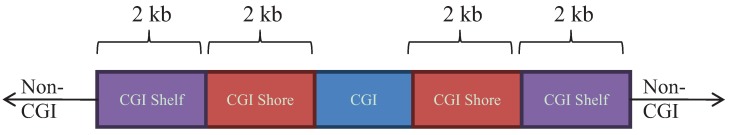
Illustration of a CpG island (CGI) with surrounding CGI shores, CGI-shelves and non-CGI regions.

CpGs that passed QC criteria (N = 420,921) were mapped to genomic features, DNase I hypersensitive sites (DHS) and transcription factor binding sites (TFBS), as described by Slieker *et al.* [7]. The genomic feature annotation is based on first assigning CpGs to one of five gene-centric regions: intergenic region (>10 kb from the nearest transcription start site (TSS)), distal promoter (−10 kb to −1.5 kb from the nearest TSS), proximal promoter (−1.5 kb to +500 bp from the nearest TSS), gene body (+500 bp to 3' end of the gene) and downstream region (3' end to +5 kb from 3' end). Next, CpGs were mapped to CGIs (CG content >50%, length >200 bp and observed/expected ratio of CpGs >0.6; locations were obtained from the UCSC genome browser [44]), CGI shore (2-kb region flanking CGI), CGI shelf (2-kb region flanking CGI shore) or non-CGI regions (Figure 1). According to the gene-annotations, 14.4% of all CpGs were located in intergenic regions, 4.7% mapped to the distal promoter, 40.4% to the proximal promoter, 38.6% to the gene body and 1.9% to the downstream region. Thirty three percent of CpGs were located within CGIs, 23.8% in shores, 9.2% in shelves and 34.0% outside CGIs. The locations of DHS and TFBS, which were described by the ENCODE project [3], were downloaded from the UCSC genome browser. Finally, CpGs were mapped to imprinted genes that were described by Yuen *et al.* [45].

### 2.6. Statistical Analysis of Twin Data

To examine the s imilarity of DNA methylation profiles of MZ twins, we computed correlations between the normalized β-values of MZ co-twins using the following two approaches: (1) for each MZ twin pair, the Spearman correlation (rho) was computed between the β-values of Twin 1 and the β-values of Twin 2 (across all CpGs, *i.e.*, CpGs are cases), as a measure of the overall similarity of the methylation profiles of each twin pair; (2) for each CpG, the Spearman correlation (rho) was computed between the β-value of Twin 1 and the β-value of Twin 2 (across all 10 MZ twin pairs, *i.e.*, MZ twin pairs are cases), as a measure of the similarity of the methylation level of a CpG in MZ twins. For Scenario 2, we describe the range of correlations for the most variable CpGs. The most variable CpGs were additionally grouped by genomic annotations and average methylation level. For each CpG, the average methylation level (β-value) and the standard deviation (SD) were computed across subjects (20 MZ twins). Based on the average β, CpGs were classified as hypomethylated (mean β < 0.3), intermediately methylated (mean β ≥ 0.3–0.7), or hypermethylated (mean β ≥ 0.7). Based on the SD, CpGs were classified as “most variable CpGs” if they had an SD ≥ 0.05.

## 3. Results and Discussion

### 3.1. DNA Methylation Level across the Genome

**Figure 2:**
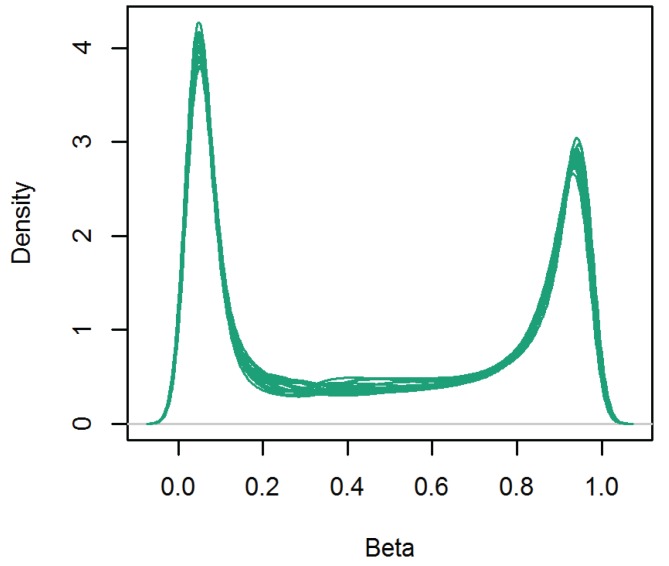
Density of β-values after normalization for all twin samples.

**Figure 3:**
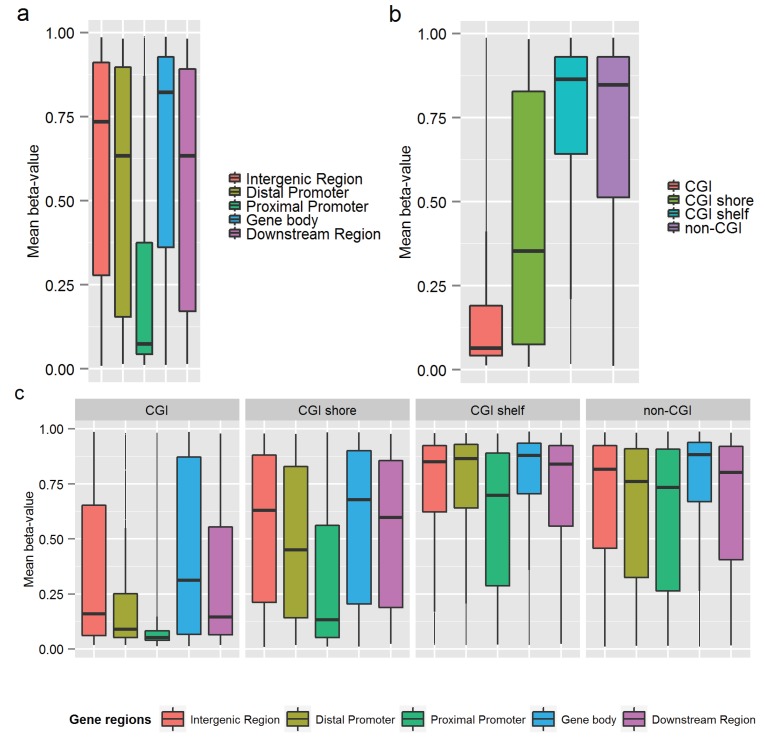
Average methylation level of individual CpGs across gene regions (**a**), CpG islands (CGI) and non-CGI regions (**b**) and for each genomic feature separately (**c**).

After QC of the methylation data, 420,921 CpGs from 10 monozygotic twin pairs were analyzed. The methylation level across genome-wide CpGs showed the typical bimodal distribution for each subject (Figure 2). Based on our β-value cut-offs (see the “Experimental” section); 184,765 CpGs (43.9%) were classified as hypomethylated, 64,829 CpGs (15.4%) were intermediately methylated and 171,327 CpGs (40.7%) were hypermethylated. CGIs were on average hypomethylated, with CGIs in proximal promoter regions showing a narrow range of average methylation levels across individual CpGs and CGIs in gene bodies, downstream regions and intergenic regions showing a broader range of methylation levels across individual CpGs (see Figure 3). Compared to CGIs, the shores, shelves and non-CGI regions on average had a higher methylation level, except for proximal promoter shores. Shores generally showed the widest range of average methylation levels across individual CpGs, when compared to CGIs, shelves and non-CGI regions (Figure 3).

### 3.2. Similarity of Genome-Wide Methylation Profiles of MZ Twins

**Figure 4:**
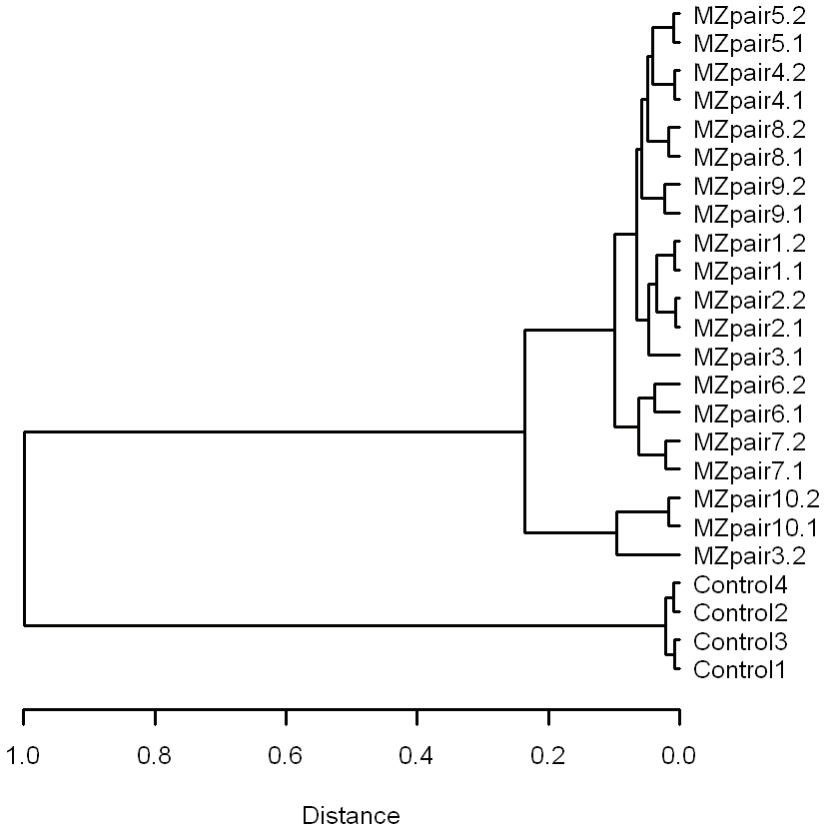
Cluster dendrogram of all twin and control samples. From left to right, the first two branches separate the control samples (HapMap cell line DNA) from the buccal samples from twins.

A cluster analysis of the methylation data revealed that all but one MZ twin clustered closely together with their co-twin (Figure 4), which could be related to differences in the cellular composition of the samples of this twin pair. Buccal swab samples are mainly composed of buccal epithelial cells with a small proportion of leukocytes, but the exact proportions may vary between persons. Using information from a reference 450k methylation dataset [7], we examined potential variation between twin samples in the proportion of buccal *versus* blood cells, by clustering the twin data based on methylation values at CpGs that showed a large difference in methylation between blood and buccal samples in the reference dataset (see the Supplementary Methods). Although some variation was indicated by this approach, the exclusion of twin samples with putatively deviant cellular proportions yielded similar results for the correlation analyses (see Table S1 and Figure S21), and we therefore decided to keep all samples in the analyses reported in this paper.

**Figure 5:**
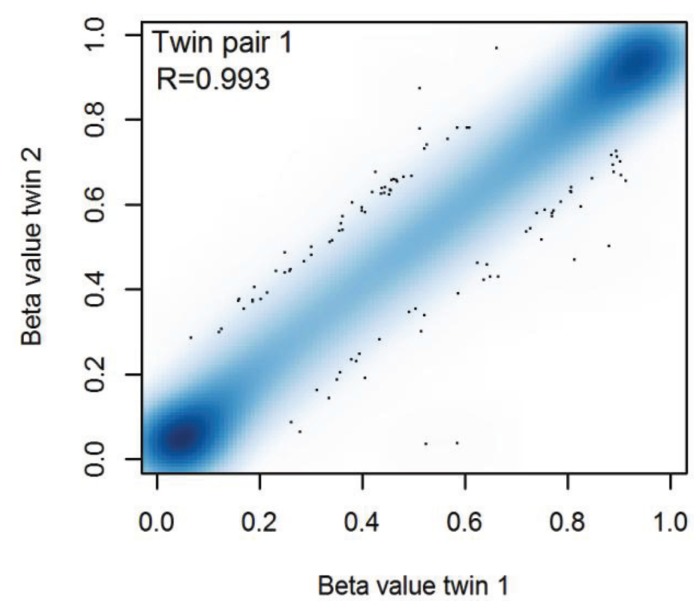
Smooth scatterplot of DNA methylation levels (β-values) at 420,921 CpGs in buccal cells from a monozygotic twin pair.

Figure 5 shows a typical scatterplot of genome-wide CpG methylation levels in buccal cells from an MZ twin pair. It illustrates that overall, the buccal DNA methylation profiles of MZ pairs are highly concordant when all CpGs are considered (rho = 0.981–0.994 for different MZ pairs, mean rho = 0.991); however, these correlations are to a large extent driven by invariable CpGs that are hypomethylated or hypermethylated in both twins. For pairs of unrelated subjects, the mean correlation was 0.983 (range: 0.970–0.992). When comparing only the most variable CpGs (SD of β ≥ 0.05), the correlations ranged from 0.869 to 0.989 (mean rho = 0.966) in MZ twins (and mean rho = 0.859, range: 0.608–0.963 for unrelated subjects). Thus, when looking only at CpGs that may vary between individuals, the overall pattern of methylation across CpGs is still highly similar within MZ pairs on average, but more variation between individual pairs becomes visible, as the methylation level at variable CpGs overall was more strongly correlated for some MZ pairs than for others. This finding is in line with the results from Martino *et al.* based on buccal cells from twins at birth and at the age of 18 months [22], which also indicated that some MZ pairs are more similar than other pairs with respect to their DNA methylation profiles.

### 3.3. Similarity of the Methylation Level at Individual CpGs in MZ Twins

Although all ten MZ twin pairs showed high overall similarity of methylation across genome-wide CpGs, some CpGs differed within MZ twin pairs (Figure 5), and we questioned how similar the methylation level at individual CpGs is when summarized across all MZ pairs. To this end, we computed for each CpG the correlation between methylation values of MZ twins. A high MZ twin correlation for a CpG suggests that MZ co-twins consistently show similar methylation levels at this CpG, indicating little stochastic and environmental variation (including measurement error) at this site, whereas a low MZ twin correlation for a CpG suggests dissimilar methylation levels in co-twins, which is indicative of a large degree of stochastic and environmental influences. 

Summarizing the individual CpG correlations over all 420,921 CpGs, the average MZ twin correlation was 0.31 (median = 0.35, range: −0.963–1), which is in line with the low heritability across genome-wide CpGs reported by previous studies [21,33,34]. However, as the majority of CpGs showed very little variation in the methylation level between subjects, all subsequent analyses were conducted using only the most variable sites (N = 59,041), which showed an average genome-wide correlation of 0.54 (median = 0.54, range: −0.661–1) in MZ twins. These findings suggest that while the large majority of CpGs are either hyper- or hypo-methylated and show little between-individual variation in DNA methylation in buccal samples, a small portion does vary markedly, and these CpGs are on average moderately to strongly correlated in MZ twins.

**Figure 6:**
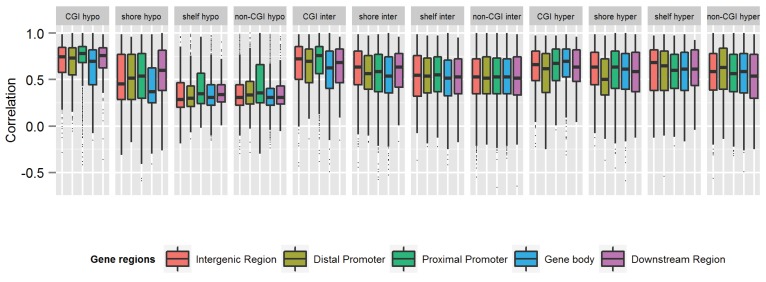
MZ twin correlations for individual CpGs grouped by genomic region and average methylation level. Hypo = Hypomethylated. Inter = intermediate methylation. Hyper = Hypermethylated. Results are based on the most variable CpGs (N = 59,041).

Table 1 describes the MZ twin correlations separately for various genomic regions and separately for hypomethylated, intermediately methylated and hypermethylated CpGs. Comparing the different gene-centric classifications, the average MZ twin correlation was highest for CpGs in proximal promoter areas (mean rho = 0.57) and lowest for gene body CpGs (mean rho = 0.51). The MZ twin correlation of methylation values was also lower on average in CGI shores (mean rho = 0.54), shelves (mean rho = 0.50) and non-CGI regions (mean rho = 0.49) compared to CGIs (mean rho = 0.66). Looking at the MZ twin correlations across genome annotations separately for hypomethylated (29.8% of variable CpGs), intermediately methylated (50.0% of variable CpGs) and hypermethylated CpGs (20.2% of variable CpGs), the median MZ twin correlation was consistently lower in the shelves, shores and non-CGI regions compared to CGIs, for all genic and intergenic regions, and this difference was most pronounced for hypomethylated CpGs (Figure 6). This observation suggests that the relative influence of familial *versus* individual-specific influences differs between these regions, with regions of low CpG density showing more variation due to individual-specific environmental and stochastic factors compared to CpG dense regions. Larger methylation discordance of MZ twins in CGI shores and shelves was also previously indicated by studies of neonatal twins [21,22]. Our results thus replicate previous findings and add to these findings that the pattern previously observed in MZ twins at birth is also visible in childhood and adolescence.

**Table 1:** Spearman correlation between the methylation values of monozygotic (MZ) twins for individual CpGs. Results are based on the most variable CpGs (N = 59,041).

Category	N CpGs	Mean rho	Median rho	Min rho	Max rho
All CpGs	59,041	0.54	0.54	−0.661	1
Gene-centric annotations	N CpGs (%)	Mean rho	Median rho	Min rho	Max rho
Intergenic (>10 kb from TSS)	11,430 (19.4%)	0.52	0.53	−0.56	1
Distal Promoter (−10 kb to −1.5 kb from TSS)	3193 (5.4%)	0.53	0.53	−0.54	1
Proximal Promoter (−1.5 kb to +500 bp from TSS)	17,880 (30.3%)	0.57	0.62	−0.66	1
Gene Body (+500 bp to 3' end)	25,163 (42.6%)	0.51	0.50	−0.59	1
Downstream region (3' end to +5 kb from 3' end)	1375 (2.3%)	0.55	0.55	−0.66	1
CGI annotations	N CpGs (%)	Mean rho	Median rho	Min rho	Max rho
CGI	10,576 (17.9%)	0.66	0.73	−0.49	1
CGI shore	14,803 (25.1%)	0.54	0.55	−0.59	1
CGI shelf	6001 (10.2%)	0.50	0.49	−0.54	1
Non-CGI	27,661 (46.9%)	0.49	0.47	−0.66	1
Methylation level	N CpGs (%)	Mean rho	Median rho	Min rho	Max rho
Hypomethylated (average beta <0.3)	17,581 (29.8)	0.48	0.42	−0.59	1
Intermediately methylated (average beta ≥0.3–0.7)	29,519 (50.0)	0.55	0.56	−0.66	1
Hypermethylated (average beta ≥0.7)	11,941 (20.2)	0.58	0.61	−0.59	1

The most strongly correlated CpGs in MZ twins (mean rho = 0.73) were hypomethylated CpGs located in proximal promoter CGIs (N = 2547 CpGs, constituting 4.3% of the most variable CpGs, and 2.9% of all CpGs located in proximal promoter CGIs), while MZ twin correlations on average were lowest in hypomethylated non-CGI gene body CpGs (N = 2972 CpGs constituting 5.0% of the most variable CpGs, Mean rho = 0.34). In combination with our observation that most proximal promoter CpGs are on average hypomethylated (Figure 3), these findings indicate that DNA methylation variation is generally depleted in proximal promoter CGIs. Yet, a small proportion of CpGs in proximal promoter CGIs does show marked variation in young and adolescent individuals, and the high average MZ twin correlations at these sites suggest that this variation may be to a large extent under genetic control.

### 3.4. MZ Twin Resemblance at CpGs in ENCODE Regulatory Regions

To further examine DNA methylation at regulatory regions in the genome, we focused specifically on CpGs located within DNase I hypersensitive sites (DHS) and CpGs within transcription factor binding sites (TFBS) identified by the ENCODE project. It has previously been described that these regions are enriched among disease-associated genetic variants [3], but it has not yet been studied to which extent heritable *versus* other sources of variation account for variation in DNA methylation in these regions. We found that both DHS and TFBS were on average hypomethylated, as expected for transcriptionally active DNA (DHS: mean β = 0.27, median = 0.09; TFBS: mean β = 0.24, median = 0.08). The most variable CpGs in these areas (representing 16.2% of all CpGs in DHS and 13.7% of CpGs in TFBS) showed a mean correlation of 0.52 (DHS) and 0.53 (TFBS), respectively, in MZ twins. These results suggest that buccal cells overall show little variation in the methylation level at the majority of CpGs within DHS and TFBS. A small proportion of CpGs in DHS and TFBS, however, does show variation between individuals, and these sites were moderately to strongly correlated in MZ twins, suggesting that these sites may be of particular interest for follow-up in future studies of heritability.

### 3.5. MZ Twin Resemblance at CpGs in Imprinted Genes

At imprinted genes, one of the alleles is typically methylated to repress expression, while the other allele is unmethylated, depending on the parent from whom the allele was inherited. This results in a methylation level of around 50% at imprinted CpGs when the two alleles are measured simultaneously. A previous twin study demonstrated moderate to high heritability at CpGs at two imprinted loci [29], suggesting that CpGs within imprinted genes may on average show more heritable variation compared to most other genome-wide CpGs. In our dataset, 346 CpGs were located in DMRs (differentially methylated regions) of 59 imprinted genes, described by Yuen *et al.* [45]. These genes were identified as imprinted in human placental tissue, and although some of these genes showed similar methylation patterns in one or multiple fetal tissues, including muscle, brain and kidney, it is unknown whether these genes are also imprinted in buccal cells. From the Yuen *et al.* set, 144 CpGs in 46 genes (see Table S2) showed a methylation level indicative of imprinting in our data (intermediate methylation; mean β ≥ 0.3–0.7). The average MZ twin correlation for this set of CpGs was 0.47 (median rho = 0.50), suggesting that MZ twin correlations at imprinted gene CpGs on average are comparable to the MZ twin correlation at intermediately methylated CpGs in general. 

### 3.6. Interpretation and Future Directions

The average twin correlation of methylation values for MZ twins at individual CpGs was low across all measured genome-wide CpGs, but it was moderate to large on average when focusing only on variably methylated CpGs. This is in line with results from a heritability analysis of DNA methylation in adipose tissue, which showed that the average heritability across all CpGs was higher for the top 10% of CpGs with the largest standard deviation of methylation level across subjects [34].

Importantly, in addition to the effects of environmental and stochastic influences, differences in DNA methylation within MZ twin pairs may result from variation in the cellular composition of samples and from technical variation (including measurement error). Buccal swab samples are mainly composed of buccal epithelial cells with a small proportion of leukocytes, but the exact proportions may vary between persons, which could lead to methylation variation within MZ twin pairs that mainly tag differences in cell type composition. We examined the impact of variation in the proportion of buccal *versus* leukocytes on our data by studying the methylation patterns of all twin samples at CpGs with a large methylation level difference between buccal and blood samples (see Supplementary Methods). Exclusion of four twin pairs, for which this approach indicated a more deviant cellular composition in one or both twins (lower proportion of buccal epithelial cells; see Figure S2 and Table S3), however, had very little impact on the average MZ twin correlations reported in this paper and led to the same conclusions (See Table S1 and Figure S1 for the results based on the exclusion of the putatively more heterogeneous samples).

With respect to technical variation, it is important to note that if the actual methylation status at a particular site is either completely unmethylated (0%) or completely methylated (100%) without true biological variation between subjects, some variability between the measured values of individuals is expected due to technical variation [46]. It is therefore likely that at sites that were on average hypomethylated or hypermethylated in our data, technical variation may account for a large part of the observed variation (although true biological variation may of course also account for part of the variation at these sites). An interesting question that largely remains to be examined is what types of environmental influences can induce changes in DNA methylation and thereby possibly impact on gene expression. Although our study design does not provide insight with regard to which of the observed differences between twins are the result of different environmental exposures and which differences have arisen due to stochastic variation in molecular processes, future studies of MZ twins who are discordant for environmental exposures should allow one to examine the effects of such influences on DNA methylation. Our finding that many CpGs in the genome show dissimilar methylation levels in young and adolescent MZ twins indicates that it is of interest for further studies to specifically search for regions in the genome where differential methylation in MZ twin coincides with differential exposures. As we observed that DNA methylation in MZ twins is overall less similar at CpGs in non-CGI regions, CGI shores and shelves, these regions are of particular interest to studies examining environmental exposures, as these regions may show the strongest effects of environmental influences.

To check whether the lower average MZ twin correlation at hypomethylated sites is not merely related to the distribution of β-values being truncated at zero (and one) by definition, we also ran the analyses on M-values (M = 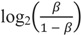), which have better statistical properties, but reduced biological interpretability, compared to β-values [47]. The MZ twin correlations based on M-values were highly similar to those based on β-values and showed a similar genome-wide average (Table S4) and a similar pattern across regions and mean methylation categories (Figure S3). Irrespective of whether the lower resemblance of MZ twins mainly reflects that these sites harbor more biological variation that is unique to MZ twins or reflects that more variation at these sites is related to measurement error, our findings provide useful information for future heritability and mQTL studies. CpGs that are very weakly correlated between MZ twins are not likely to show high heritability or strong effects of DNA variants on the methylation level.

A limitation of our study is the modest study size, which limited the scope of our analyses to the description of the major patterns (*i.e.*, averages) of twin correlations across the genome. A second limitation is that we did not include DZ twins. The correlation between the phenotypes of MZ twins summarizes the contribution of heritable influences and shared environmental factors to phenotypic variation. It thus remains to be established whether CpGs that were strongly correlated in MZ twin pairs are strongly affected by heritable influences or whether shared environmental influences are also important at these sites. Of interest, a previous twin study of DNA methylation in adipose tissue identified a number of CpGs with evidence for shared environmental effects on DNA methylation [34]. Future studies that include data from both MZ and DZ twin pairs are needed to separate the effects of heritable effects and shared environment on genome-wide DNA methylation profiles in buccal cells. Our results indicate that such studies are worthwhile, as we have shown that methylation at a number of CpGs is strongly correlated between MZ twins in buccal cells.

We studied DNA methylation extracted from buccal samples, which may be easier to collect than blood samples in, e.g., young children, and are therefore well-suited for large-scale studies in humans. A relevant question is how representative DNA methylation extracted from these samples is for DNA methylation variation in other tissues and whether methylation studies of buccal *vs.* blood-derived DNA would lead to similar insights. Although DNA methylation patterns are to a large extent tissue-specific [7] and epigenetic changes arising later in life in one tissue may not be detectable in others, epigenetic variation that is established early in development is more likely to be reflected in multiple tissues [4]. Yet, the methylation patterns of buccal cells are likely to be more informative to the methylation state of other ectoderm-derived tissues, whereas methylation patterns in blood may be more comparable to other mesoderm-derived tissues. Finally, it may be regarded as an advantage that compared to blood, which consists of many different cell types, buccal samples represent a relatively homogenous sample type [48], in the sense that it consists of only two major cell types, which potentially makes correction for cell types more straightforward. On the other hand, an advantage of blood samples is that they may provide more insight into DNA methylation variation related to immune system-mediated processes in the body, which are important in many diseases. To conclude, blood and buccal samples are both valuable for gaining insight into the overall importance of heritable and environmental factors to DNA methylation variation in the genome, and our study showed that the average genome-wide MZ twin correlation for DNA methylation in buccal cells is similar to the average correlation previously reported for peripheral blood [33]. 

## 4. Conclusions

To summarize, we computed genome-wide MZ twin correlations for the buccal DNA methylation level at individual CpGs. Methylation levels in MZ twins were moderately to strongly correlated at CpGs with the largest inter-individual variation, which constituted a relatively small proportion of the CpGs that were measured. The average MZ twin correlation across all CpGs was relatively low (mean rho = 0.31), which is similar to findings from previous twin studies [21,33]. Although most CpGs within CGIs were on average hypomethylated, some of them showed large variation in methylation levels. We observed that CpGs with variable methylation levels were more strongly correlated in MZ twins when located in CGIs compared to CpGs in shores and shelves. CpGs in DHS and TFBS were generally hypomethylated, as expected for regulatory active DNA, but CpGs in these regions that were more variably methylated were moderately to strongly correlated in MZ twin pairs, in line with our findings for variably methylated CpGs in general. To conclude, we have shown that in buccal samples from young and adolescent MZ twins, most CpGs show an average methylation level close to zero or 100% and little inter-individual variation, and a subset of CpGs show larger variability with evidence for a familial component (DNA sequence variation or shared environment). These findings are relevant for future heritability studies of DNA methylation and for mQTL studies.

## Supplementary Files

Supplementary File 1 Supplementary Information (PDF, 294 KB)
