# Supplementary material for: Epigenetic Variation in Monozygotic Twins: A Genome-Wide Analysis of DNA Methylation in Buccal Cells
Source: Genes (Basel). 2014 May 5;5(2):347–65. doi: 10.3390/genes5020347 (PMC4094937; doi:10.3390/genes5020347)
Supplement: Supplementary File 1 — Supplementary Information (PDF, 294 KB) [file genes-05-00347-s001.pdf]

Supplementary Information

Supplementary Methods

To gain insight into the degree to which our data may be influenced by variation in the cellular composition of buccal swab samples, we first looked at the beta-values of our twin samples at cg18384097; a CpG site located in the *PTPN7* (protein tyrosine phosphatase non-receptor type 7) gene. Based on a comparison of publicly available DNA methylation data from blood samples and buccal swab samples [1–3], cg18384097 was found to be one of the most differentially methylated CpGs between buccal swabs samples and blood (beta-value buccal = 0.82 and beta-value blood = 0.05), suggesting that this CpG could be a suitable marker to obtain insight into the relative proportions of buccal *versus* blood cells within a sample [4]. In our buccal data from twins, the average beta-value of this CpG was 0.89. Beta-values for this CpG for all twin samples are presented in Table S3. Table S3 indicates that the majority of twins had similar methylation levels at this CpG, although some variation is evident. MZ twin 3.2 had the most deviant methylation value, which suggests that the sample of this individual may have contained relatively more blood cells compared to the other samples.

To examine this further, we next turned to another reference dataset consisting of 450k methylation data on multiple tissue types [5] and selected from this dataset all CpGs with an average beta-value difference >0.6 between buccal swab samples (N = 5) and blood samples (N = 5), in order to obtain a set of CpGs of which the methylation value measured in buccal swab samples is presumably reflective of the amount of buccal epithelial cells *versus* blood cells present in buccal swab samples. This selection yielded 881 CpGs (p-value range: 2.58×10^{-5}–7.15×10^{-4}), after excluding probes containing SNPs in the CpG site. For this set of 881 CpGs, we plotted the methylation beta-values of our buccal samples from twins together with beta-values from the reference set [5] for buccal swab samples (N = 5), blood samples (N = 5) and saliva samples (N = 5) in a heatmap (Figure S2). Figure S2 illustrates that some variation in methylation level at these CpGs is present between the buccal swab samples: some buccal samples show relatively more intermediate methylation, a pattern that is more similar to the saliva reference samples, and is suggestive of a higher proportion of blood cells in the sample. Based on methylation levels at this set of CpGs that differentiates strongly between buccal and blood samples, two smaller clusters were identified among buccal swab samples, containing twin Samples 3.2, 6.2, 7.1, 10.1 and 10.2 and reference buccal Samples 3 and 5 (Figure S2), which demonstrated more intermediate methylation values compared to the other buccal samples, suggesting that the samples in the two small clusters (twin Samples, 3.2, 6.2, 7.1, 10.1 and 10.2) contained higher proportions of leukocytes compared to the other buccal samples. Twin Samples, 3.2, 6.2, 7.1, 10.1 and 10.2 also showed the lowest methylation beta-values at cg18384097 (Table S3). To examine the extent to which our analyses may be affected by heterogeneity across samples related to cell type proportions, we repeated our analyses with twin Pairs 3, 6, 7 and 10 excluded, thus keeping only the most homogenous samples that seemed to have the highest buccal epithelial cell content (based on the approach illustrated in Figure S2) in the analyses, which yielded highly similar results (see Table S1 and Figure S1).

Table S1. Correlations between methylation values of twins, with twin Pairs 3, 6, 7 and 10 excluded.

Category	N CpGs	Mean rho	Median rho	Min rho	Max rho
All CpGs	59,041	0.57	0.60	−1	1
Gene-centric annotations	N CpGs (%)	Mean rho	Median rho	Min rho	Max rho
Intergenic (>10 kb from TSS)	11,430 (19.4%)	0.56	0.60	−1	1
Distal Promoter (−10 kb to −1.5 kb from TSS)	3193 (5.4%)	0.57	0.60	−0.89	1
Proximal Promoter (−1.5 kb to +500 bp from TSS)	17,880 (30.3%)	0.60	0.66	−0.94	1
Gene Body (+500 bp to 3' end)	25,163 (42.6%)	0.56	0.60	−1	1
Downstream region (3' end to +5 kb from 3' end)	1375 (2.3%)	0.58	0.66	−0.94	1
CGI annotations	N CpGs (%)	Mean rho	Median rho	Min rho	Max rho
CGI	10,576 (17.9%)	0.66	0.77	−1	1
CGI shore	14,803 (25.1%)	0.57	0.60	−0.89	1
CGI shelf	6001 (10.2%)	0.55	0.60	−1	1
Non-CGI	27,661 (46.9%)	0.55	0.60	−1	1
Methylation level	N CpGs (%)	Mean rho	Median rho	Min rho	Max rho
Hypomethylated (average beta <0.3)	17,581 (29.8)	0.58	0.60	−0.94	1
Intermediately methylated (average beta ≥0.3–0.7)	29,519 (50.0)	0.58	0.60	−1	1
Hypermethylated (average beta ≥0.7)	11,941 (20.2)	0.54	0.60	−1	1

Figure S1. MZ twin correlations for individual CpGs grouped by genomic regions and average methylation level, with twin Pairs 3, 6, 7 and 10 excluded. Hypo = hypomethylated. Inter = intermediate methylation. Hyper = hypermethylated.

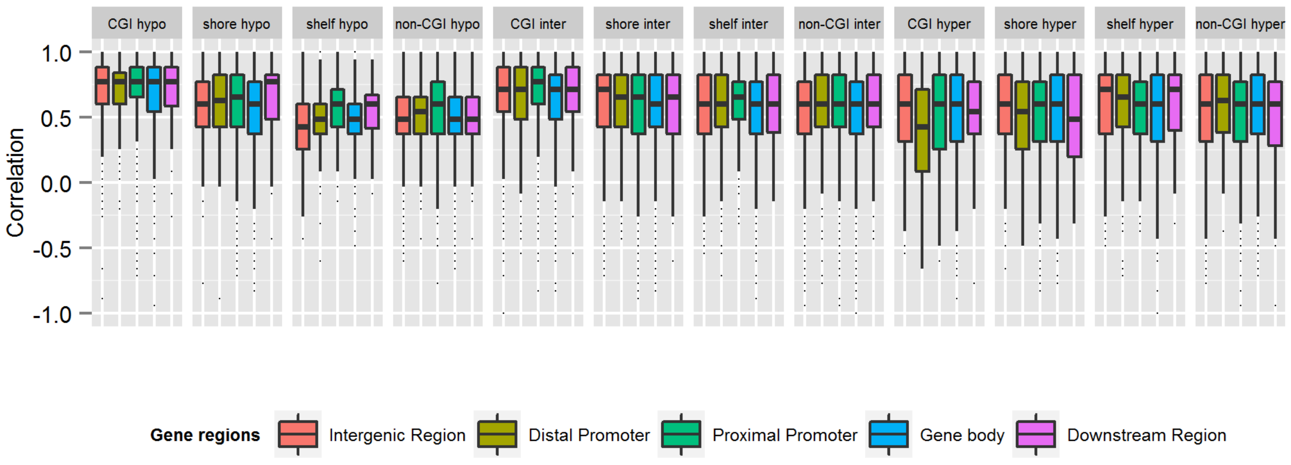

Table S2. Names and number of analyzed CpGs of imprinted genes from Yuen *et al*. [6].

Gene No.	Gene Name	N CpGs analyzed [A]	Gene No.	Gene Name	N CpGs analyzed [A]
1	*ABCA1*	1	24	*NNAT*	3
2	*ANKRD11*	1	25	*OSBPL5*	1
3	*ATP10A*	4	26	*PEG10*	5
4	*CALCR*	5	27	*PEG3*	1
5	*CDKN1C*	1	28	*PHLDA2*	7
6	*COPG2*	1	29	*PLAGL1*	3
7	*DDC*	2	30	*PPP1R9A*	2
8	*DLX5*	4	31	*PRIM2A*	3
9	*GNAS*	17	32	*RBP5*	1
10	*GRB10*	7	33	*SGCE*	2
11	*H19*	4	34	*SLC22A18*	1
12	*IGF2*	1	35	*SLC22A18AS*	1
13	*IGF2AS*	1	36	*SLC22A2*	4
14	*IGF2R*	2	37	*SLC22A3*	1
15	*INPP5F*	1	38	*SNRPN*	2
16	*KCNQ1*	1	39	*SNURF*	6
17	*KCNQ1DN*	1	40	*TCEB3C*	1
18	*KLF14*	9	41	*TFPI2*	5
19	*L3MBTL*	2	42	*TP73*	5
20	*MAGEL2*	1	43	*UBE3A*	4
21	*MEG3*	3	44	*WT1*	3
22	*MEST*	2	45	*ZIM2*	6
23	*NDN*	2	46	*ZNF264*	4
				Total:	144

[A] The number of CpGs in each of the imprinted genes described by Yuen *et al.*, which showed an intermediate methylation level in our buccal data from twins (mean $\beta \geq 0.3$–0.7 across subjects).

Figure S2. Beta-values of twin samples (buccal swabs) and reference data from five blood samples, five saliva samples and five buccal samples for CpGs with an average (absolute) beta-value difference >0.6 between blood and buccal in the reference data. Brightest yellow: beta-value = 1. Brightest blue: beta-value = 0. The clustering of CpGs (N = 881, y-axis) and samples (x-axis) was performed using complete linkage based on the Euclidian distance between beta-values.

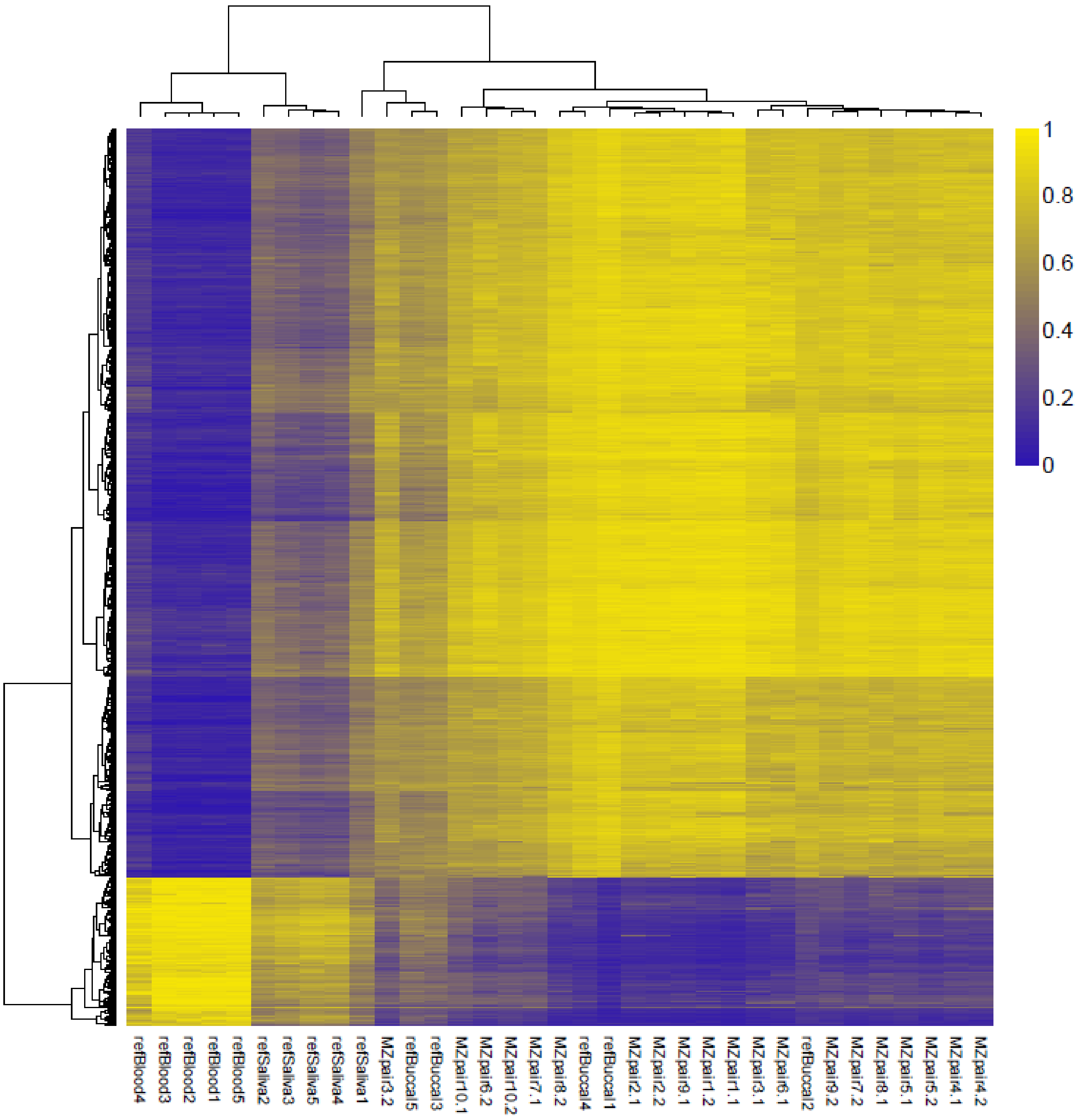

Table S3. Beta-values of twin samples for cg18384097 in the in *PTPN7* gene; a CpG that was previously reported to be highly discriminative between blood samples and buccal swab samples [1–4].

Sample	Beta-value cg18384097
MZpair3.2	0.7434
MZpair10.2	0.8360
MZpair6.2	0.8513
MZpair7.1	0.8527
MZpair10.1	0.8583
MZpair9.1	0.8723
MZpair7.2	0.8751
MZpair4.2	0.8929
MZpair5.1	0.8949
MZpair9.2	0.9002
MZpair8.2	0.9037
MZpair2.1	0.9055
MZpair1.2	0.9057
MZpair6.1	0.9076
MZpair5.2	0.9129
MZpair3.1	0.9130
MZpair4.1	0.9165
MZpair2.2	0.9187
MZpair8.1	0.9230
MZpair1.1	0.9312
**Average**	**0.8857**

Highlighted in blue are the twin samples that clustered separately from the other twin samples in Figure S2. The samples in the table are sorted by beta-value (lowest to highest).

Table S4. Spearman correlation between the methylation level of MZ twins at individual CpGs based on M-values.

Genome-wide	N CpGs	Mean r	Median r	Min r	Max r
All variable CpGs	59,041	0.54	0.54	−0.66	1

Figure S3. MZ twin correlations for individual CpGs grouped by genomic region and average methylation level, based on M-values. Hypo = hypomethylated. Inter = intermediate methylation. Hyper = hypermethylated.

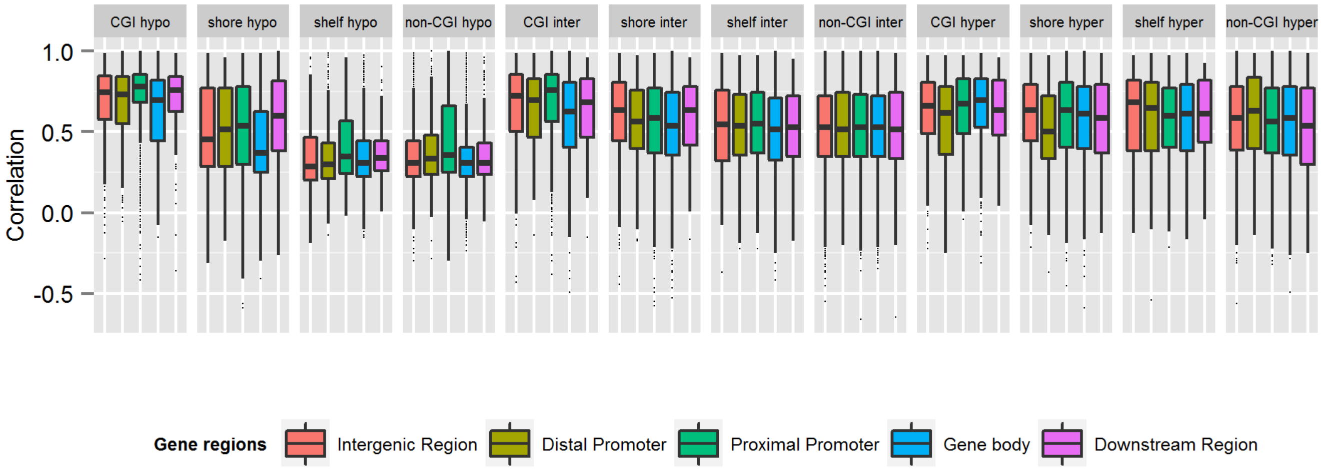

References

1. Calvanese, V.; Fernandez, A.F.; Urdinguio, R.G.; Suarez-Alvarez, B.; Mangas, C.; Perez-Garcia, V.; Bueno, C.; Montes, R.; Ramos-Mejia, V.; Martinez-Camblor, P.; *et al*. A promoter DNA demethylation landscape of human hematopoietic differentiation. *Nucl. Acids Res.* **2012**, *40*, 116–131.
2. Essex, M.J.; Boyce, W.T.; Hertzman, C.; Lam, L.L.; Armstrong, J.M.; Neumann, S.M.; Kobor, M.S. Epigenetic vestiges of early developmental adversity: childhood stress exposure and DNA methylation in adolescence. *Child Dev.* **2013**, *84*, 58–75.
3. Teschendorff, A.E.; Menon, U.; Gentry-Maharaj, A.; Ramus, S.J.; Weisenberger, D.J.; Shen, H.; Campan, M.; Noushmehr, H.; Bell, C.G.; Maxwell, A.P.; *et al*. Age-dependent DNA methylation of genes that are suppressed in stem cells is a hallmark of cancer. *Genome Res.* **2010**, *20*, 440–446.
4. Souren, N.Y.; Lutsik, P.; Gasparoni, G.; Tierling, S.; Gries, J.; Riemenschneider, M.; Fryns, J.P.; Derom, C.; Zeegers, M.P.; Walter, J. Adult monozygotic twins discordant for intra-uterine growth have indistinguishable genome-wide DNA methylation profiles. *Genome Biol.* **2013**, doi:10.1186/gb-2013-14-5-r44.
5. Slieker, R.C.; Bos, S.D.; Goeman, J.J.; Bovee, J.V.; Talens, R.P.; van der Breggen, R.; Suchiman, H.E.; Lameijer, E.W.; Putter, H.; van den Akker, E.B.; *et al*. Identification and systematic annotation of tissue-specific differentially methylated regions using the Illumina 450k array. *Epigenetics Chromatin* **2013**, doi:10.1186/1756-8935-6-26.
6. Chen, Y.A.; Lemire, M.; Choufani, S.; Butcher, D.T.; Grafodatskaya, D.; Zanke, B.W.; Gallinger, S.; Hudson, T.J.; Weksberg, R. Discovery of cross-reactive probes and polymorphic CpGs in the Illumina Infinium HumanMethylation450 microarray. *Epigenetics* **2013**, *8*, 203–209.

© 2014 by the authors; licensee MDPI, Basel, Switzerland. This article is an open access article distributed under the terms and conditions of the Creative Commons Attribution license (http://creativecommons.org/licenses/by/3.0/).
